# Distorted Views of Biodiversity: Spatial and Temporal Bias in Species Occurrence Data

**DOI:** 10.1371/journal.pbio.1000385

**Published:** 2010-06-01

**Authors:** Elizabeth H. Boakes, Philip J. K. McGowan, Richard A. Fuller, Ding Chang-qing, Natalie E. Clark, Kim O'Connor, Georgina M. Mace

**Affiliations:** 1 Natural Environment Research Council (NERC) Centre for Population Biology, Imperial College, Berkshire, United Kingdom; 2 World Pheasant Association, Newcastle University Biology Field Station, Newcastle upon Tyne, United Kingdom; 3 School of Biological Sciences, The University of Queensland, St. Lucia, Queensland, Australia; 4 The Commonwealth Science and Industrial Research Organisation (CSIRO) Climate Adaptation Flagship and CSIRO Sustainable Ecosystems, St. Lucia, Queensland, Australia; 5 College of Biological Sciences and Biotechnology, Beijing Forestry University, Beijing, China; 6 Aquatic Department, The London Aquarium, London, United Kingdom

## Abstract

Boakes et al. compile and analyze a historical dataset of 170,000 bird sightings over two centuries and show how changing trends in data gathering may confound a true picture of biodiversity change.

Historical as well as current data on species distributions are needed to track changes in biodiversity. Species distribution data are found in a variety of sources but it is likely that they include different biases towards certain time periods or places. By collating a large historical database of ∼170,000 records of species in the avian order Galliformes, dating back over two centuries and covering Europe and Asia, we investigate patterns of spatial and temporal bias in five sources of species distribution data: museum collections, scientific literature, ringing records, ornithological atlases, and website reports from “citizen scientists.” Museum data were found to provide the most comprehensive historical coverage of species' ranges but often proved extremely time-intensive to collect. Literature records have increased in their number and coverage through time, whereas ringing, atlas, and website data are almost exclusively restricted to the last few decades. Geographically, our data were biased towards Western Europe and Southeast Asia. Museums were the only data source to provide reasonably even spatial coverage across the entire study region. In the last three decades, literature data have become increasingly focussed towards threatened species and protected areas, and currently no source is providing reliable baseline information—a role once filled by museum collections. As well as securing historical data for the future and making it available for users, the sampling biases will need to be understood and addressed if we are to obtain a true picture of biodiversity change.

## The Growing demand for Biodiversity Data

Increasing awareness and concern about the continuing loss of global biodiversity has led to much recent interest in data sources that can be used to assess changes in the status and distribution of the world's species. This information may have many applications including developing models of global species diversity and its change, designing and assessing conservation actions, and tracking progress in conserving overall biodiversity. International commitments such as the Millennium Development Goals [1] and the Convention on Biodiversity [2] call for a reduction in the rate of biodiversity loss by 2010 and therefore require data to measure such trends. For these and related purposes, biodiversity information must include more than a simple snapshot of the current status and distribution of species. Whilst recent trends in population sizes or geographical ranges over time can help track progress against biodiversity targets [3],[4], long-term trends can reveal major shifts in abundance and composition of biological communities that put the status of the present-day biota into a proper historical context [5],[6]. For example, we might attempt to restore a Caribbean reef to its state when studies of reef ecology began, say 50 years ago, but this will be far removed from its pristine condition of a few hundred years earlier, the ecosystem mechanisms of which we can only guess at [7]. If we are to preserve a record to help future scientists understand the complexities of our current ecosystems, biodiversity information must be comprehensive and not just focus on the most charismatic species or those of greatest conservation concern. Although we can use aggregated population trends such as the Living Planet Index [3] or the Red List Index [8] to approximate rates of biodiversity loss, there is no substitute for primary data [9], i.e., underlying dated records of species occurrences rather than summaries at coarser resolutions or missing some attributes attached to the original record. Species occurrence data allow us to investigate biodiversity loss in far greater detail, for example, to document patterns of range collapse over time in relation to causal processes. Access to primary data also permits new questions to be asked, for which previous summaries might not be suitable. Yet, remarkably there are few globally comprehensive sets of primary data compiled for these purposes. Such information is not always publicly available and the details of sampling biases or validation may be difficult to find.

The Global Biodiversity Information Facility (GBIF) [http://www.gbif.org], which is a portal to species locality data obtained from both museums and through observations, is the predominant international, publicly funded resource that is fully open to all users with clear data-sharing principles. There are continuing efforts to improve its coverage and comprehensiveness, but data holdings for most groups are still quite sparse, somewhat biased geographically, and by no means free of errors [10]–[13]. Another widely used set of data comes from extensive collations by conservation organisations, focussing especially on the distribution and status of terrestrial vertebrates. These include species lists for specific biomes, ecoregions, and countries (e.g., World Wildlife Fund (WWF) [www.worldwildlife.org/wildfinder]), shape files representing geographic ranges and conservation status (e.g., The International Union for Conservation of Nature (IUCN) [iucn.redlist.org/mammals], The Global Amphibian Assessment (GAA) [http://www.globalamphibians.org]), and NatureServe [http://www.natureserve.org/getData/animalData.jsp.]) and species population trends [14]. However, many of these sources under-represent certain areas, in particular the species-rich tropics [10], and owing to the trade-off between coverage and detail, they are often available only at a fairly coarse scale. None of these includes historical information prior to about 1970. Consequently, for many purposes, researchers need to refer back to the primary data sources, in particular museum holdings, the published and grey literature, and Web-based resources. But how complete and consistent are these different sources, and how easy is it to obtain the information?

## Gathering Data

The Galliformes (partridges, pheasants, and quails) are a relatively well-studied group of birds for which there is an unusually good historical record. They have had a long association with people having been hunted and their feathers used for decoration and religious symbolism over much of recent human history [15]. In modern times, they have been the focus of particular attention through being one of the most threatened avian orders [16]. Galliform species are relatively common in museums and other collections, and their distinctiveness means that they are frequently and reliably recorded by field naturalists. Our study focuses on the 127 species that occur within WWF's Palaearctic and Indo-Malay biogeographic realms (Table S1). We attempted to gather all species distribution data that could be accessed from five different sources: museum collections, literature records, banding (ringing) data, ornithological atlases, and birdwatchers' trip reports housed in online collections. For each data source, exhaustive and systematic search strategies were adopted (see Box 1).

**BOX 1. Methods**

**Museum Data**

Using Web-based searches and Roselaar [27], 377 natural history collections were identified. The 338 of these for which addresses could be obtained were contacted by e-mail or letter, requesting a list of the Galliformes in their holdings along with collection localities and dates. Non-respondents were re-contacted. Information was gathered through publicly available online databases (e.g., ORNIS) and electronic or paper catalogues sent to us by the museums or museum visits.

**Literature Data**

Literature data were added to those previously collected by McGowan [28]. Entire series of key English-language ornithological journals such as *Ibis*, *Bird Conservation International*, *Journal of the Bombay Natural History Society*, etc. were scanned for relevant information, availability allowing. Relevant Chinese literature was also scanned. Additionally, data were obtained from regional reports, personal diaries, letters, newsletters, etc. stored in the archives of BirdLife International, Cambridge, UK; the WPA, Newcastle, UK; and the Edward Grey Institute, University of Oxford, UK. (The full reference list is available from the corresponding author.)

Peer-reviewed data were defined as those from a journal listed in the 2007 JCR Science Edition.

**Ringing Data**

Eighty-three ornithological ringing groups were identified using Web-based searches and were contacted via e-mail. We recorded both capture and re-capture data.

**Atlas Data**

We digitised location data from 17 ornithological atlases (Text S2). Data from several other atlases could not be used since range of dates for the records was too wide (greater than 20 years).

**Trip Report Website Data**

We used two of the largest trip report websites, http://www.travellingbirder.com and http://www.birdtours.co.uk, extracting data from all reports from European, Asian, and North African countries. Care was taken to enter reports that featured on both websites once only.

**Georeferencing and Dating Records**

Locality descriptions were converted to geographic co-ordinates using a wide range of atlases and gazetteers, co-ordinates only being assigned if accurate to one degree (although in the majority of cases the locations were actually accurate to within 10 minutes, and this proportion increased to 73% during the period 1950–2006; Table S4). Wherever possible, localities we could not georeference ourselves were sent to regional experts. If a particular locality description matched two or more places their midpoint was taken, provided this fitted our 1-degree accuracy rule. Only records dated to within ±10 years were used in the analysis.

**Data Validation**

Georeferenced data were subject to the following checks:

1. That each data point was in the country that its locality described.
2. That each data point was within reasonable distance of the species' known historical range.
3. That each data point that identifiably came from a protected area listed in the World Database of Protected Areas [29] was indeed within that protected area.

For regions/species for which we had contacts (approximately one third of the records), data were sent to experts for informal “refereeing” to highlight dubious or missing data.

After about 1,500 person-days of data gathering by a team of 18 people, the database contained a total of 171,948 records, 148,490 (86%) of which had at least an approximate date and location associated with them (see Dataset S1, GALLIFORM: WPA Eurasian Database v 1.0; http://datadryad.org). Each record indicated the data source (museum, peer-reviewed and grey literature, atlas, ringing or Web-based ornithological trip reports), and minimally a species identity, date and location. Museum collections (35,655 records: 24%) and atlases (75,073 records: 51%) were the largest contributors to the data set. The literature information (21,270 records: 14%) and ringing databases (14,879 records: 10%) were also important. Website trip reports, a relatively recent data source, contributed to less than 1% (1,393 records) of the database.

In total, data were obtained from 121 of the 338 museums that we contacted (Tables S2 and S3). Almost half of the museums we contacted did not respond despite at least one follow-up enquiry, and there was substantial variation in the amount and format of data contributed by those that did reply. Altogether, over 50% of the records came from just six museums (Natural History Museum, London; Zoological Institute of the Russian Academy of Sciences, St Petersburg; Zoological Museum of Moscow University; Field Museum of Natural History, Chicago; American Museum of Natural History, New York; National Museum of Natural History, Leiden), a single museum (the Natural History Museum, London) contributing nearly 20% of the museum records that could be georeferenced and dated.

Museums also varied enormously in the ease with which their data could be accessed (Table S2). Rather few collections have their holdings in electronic databases, and had we been restricted to those that did, we would have lost almost 14,000 records from the largest six museums alone. Even where collections were catalogued electronically, the records were often in a format that was difficult to use. We visited some of the larger museum collections and gathered information directly from specimen labels and paper records; it was clear that many older museum specimens and their labels are deteriorating. It was not uncommon for labels to be faded and/or torn, making some impossible to decipher and giving rise to the concern that others may not be legible for very much longer. Many of the collections had suffered severe funding cutbacks and simply did not have the resources to conserve adequately all of their specimens.

Due to their large body sizes and spurs, the majority of galliform species tend not to be ringed. Additionally, many of the ringing groups kept their records on paper and were not able to send them to us; of the 83 ringing groups that we contacted, just 30 replied and only seven were able to provide us with data (Text S1). Nevertheless, we were able to access (and georeference) 14,879 ringing records.

Georeferencing (i.e., finding the latitude and longitude) of each sighting or specimen proved to be a major task, not just for the museum data (Table S4). About 10% of the data collection effort was spent researching the locations in order to map them correctly. Despite utilising the help of regional experts, we were unable to georeference 15,693 records (stemming from 6,705 unique localities): 8,916 due to being unable to find the locality description in gazetteers, 6,299 due to the locality description being too vague to assign a coordinate to within an accuracy of one degree, and 478 due to two or more places that were two or more degrees apart fitting the locality description. Despite the difficulties we encountered, the majority of records that we did georeference were accurate to within 10 minutes. We had less success at georeferencing museum records than literature records, due at least in part to difficulties in reading hand-writing on specimen labels. Older records were also harder to georeference, presumably due to changes in place names over time and to some early ornithologists failing to document the collection locality. As might be expected, localities from countries that do not use the Roman alphabet were also harder to georeference. Surprisingly, records from website trip reports were even less well georeferenced than museum records. We strongly recommend that authors who intend their observations to be of practical use to others carry a GPS and include co-ordinates as well as fuller descriptions of their bird-watching localities. We were unable to date 7% of records to within 10 years—over half of the undated records coming from museum collections.

## The Historical and Geographical Spread of Information

**Figure 1:**
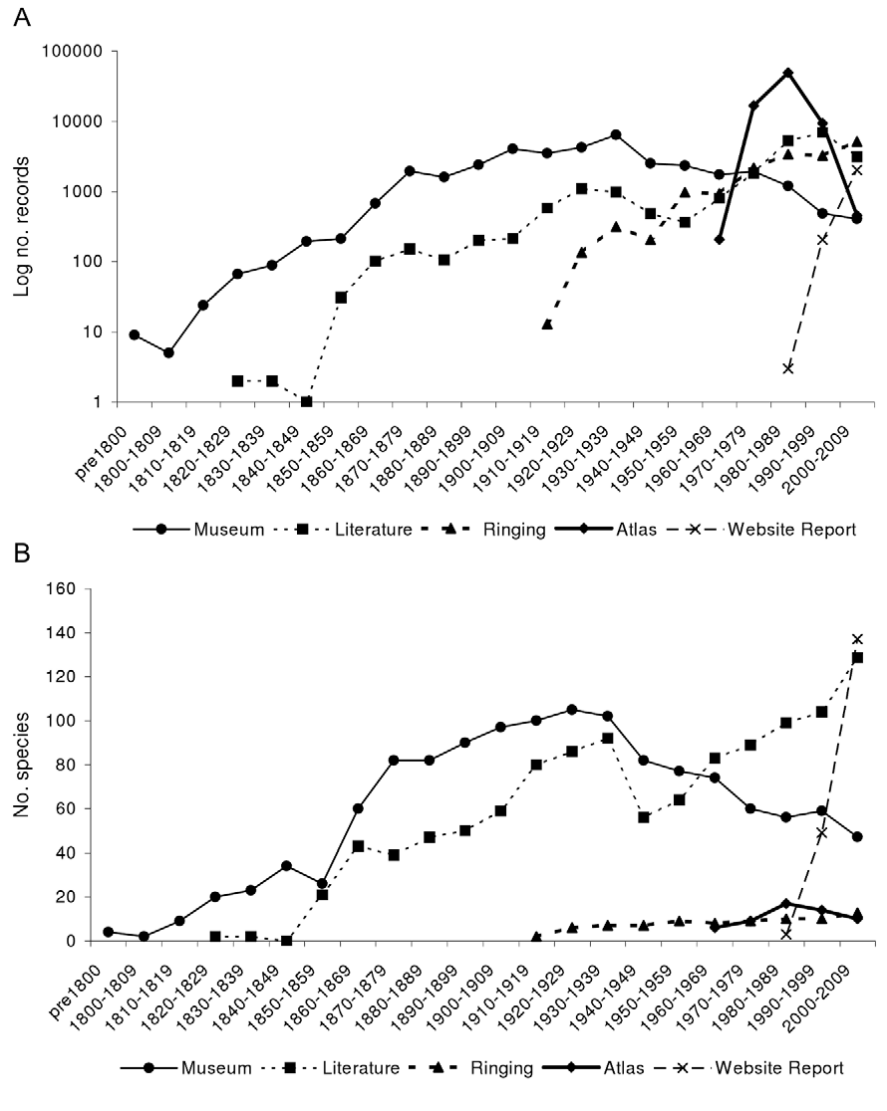
The contribution of data sources over time. A) shows the number of records contributed by each data source within each time period, and B) shows the number of species recorded by each data source within each time period. The number of records for 2000–2006 has been extrapolated to 2000–2009 for ease of comparison with the other decades.

Museum collections provide the major source of historical data (Figure 1), dominating records for periods before about 150 years ago. The quantity of information peaks during the 1930s but is not overtaken by any other source until 1970. Museum collections and literature reports contribute most to the number of distinct species recorded (Figure 1B), but the atlas records, which begin only in 1966, soon swamp the database in terms of total records (Figure 1A). Literature records generally increase through time until the 1990s although they show a dip in the period 1930–1969. From their first record in our dataset in 1911, ringing records also increase through time. The first website trip report records date from 1989 and, in line with increased accessibility of the Internet, they increase sharply from the 1990s to the current decade. Despite only contributing about 1% of records, trip reports include the highest number of species in the period 2000–2006, presumably reflecting visits by birdwatchers to regions with high species richness.

**Figure 2:**
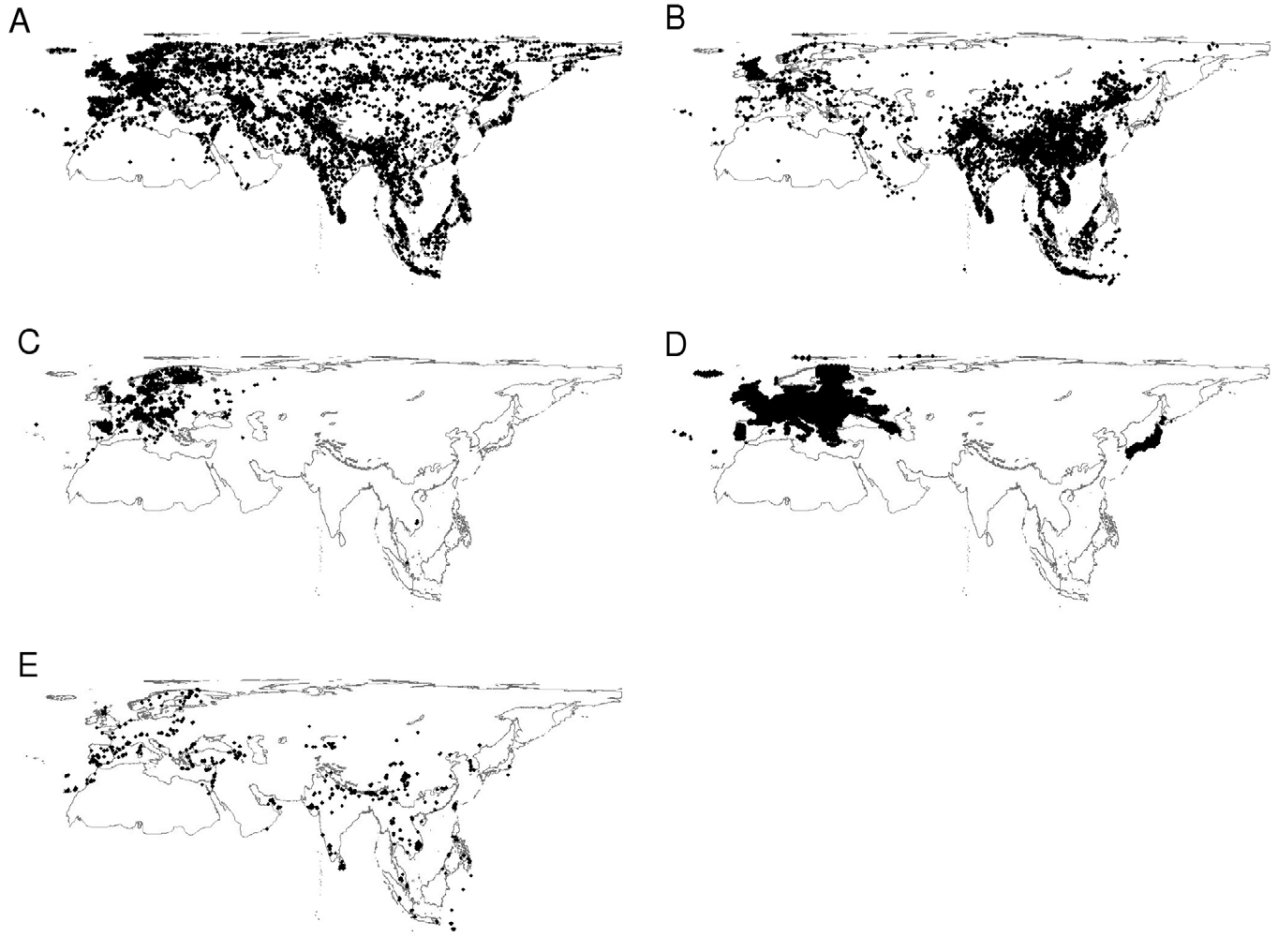
The spatial distribution of records from different sources. A) museums, B) literature, C) ringing, D) atlas, and E) website trip reports.

The distribution of records from each data source varies geographically (Figure 2) with the museum data showing the best spatial coverage. Literature records give a much denser sampling from Western Europe and China, where we had access to libraries, but a very poor sampling in Eastern Europe and Northern Asia. Ringing records tend to focus on Western Europe although this could be a reflection of the ringing stations we were able to contact. Atlases focus on Western Europe and Japan. Website report records, mainly from Western Europe and Southern Asia, probably reflect favourite locations for ornithological trips. Geographic bias may also be a result of variation in human population density and scientific capacity [17],[18].

**Figure 3:**
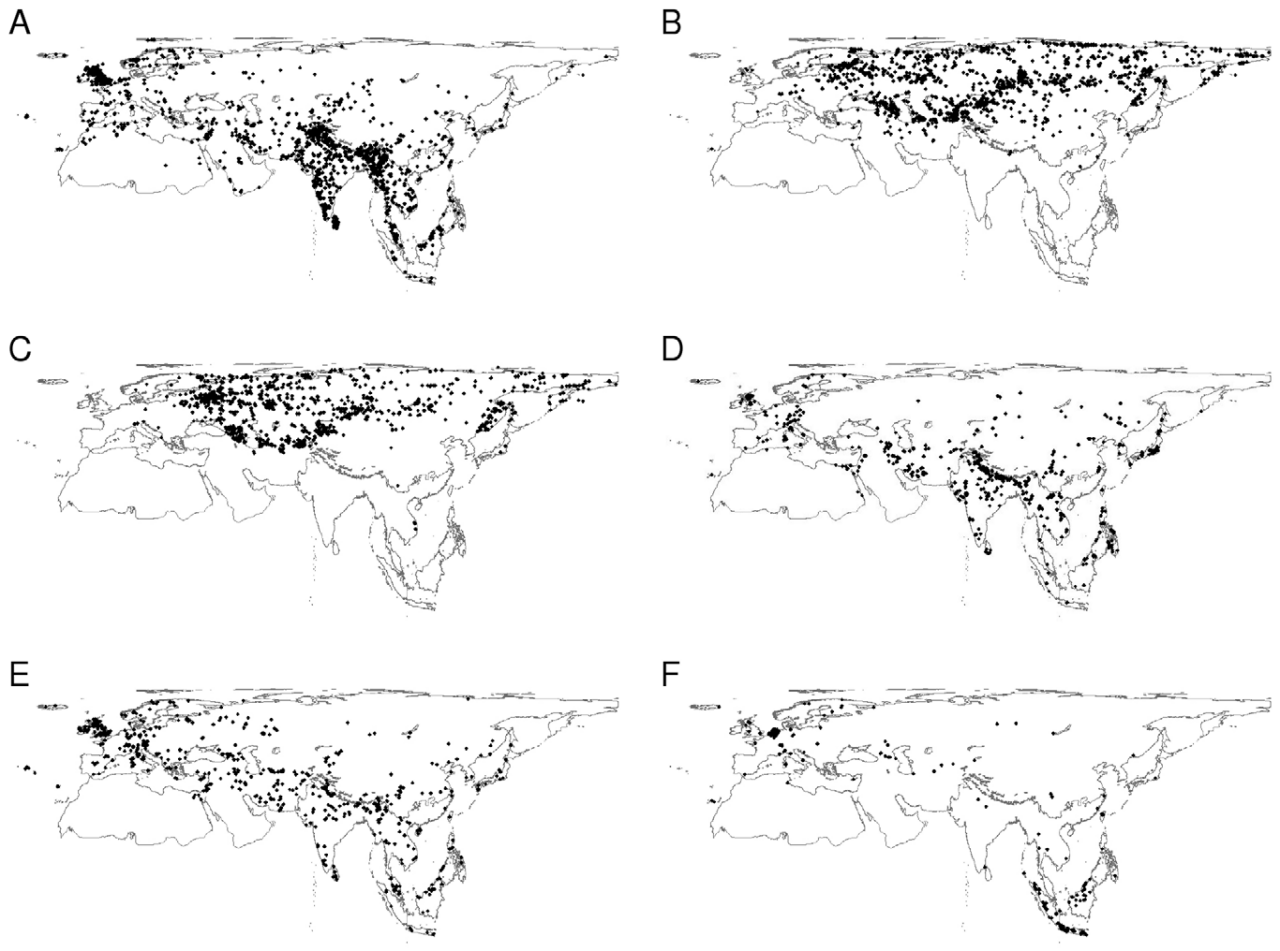
The spatial distribution of records from the six highest contributing museum collections. A) Natural History Museum, London (BMNH), B) Zoological Institute of the Russian Academy of Sciences, St. Petersburg (ZISP), C) Zoological Museum of Moscow University (ZMMU), D) Field Museum of Natural History (FMNH), E) American Museum of Natural History (AMNH), and F) National Museum of Natural History, Leiden (NNM).

Museum collections range widely in size, and the database records are dominated by large collections in the major, national museums which tend to have a specific geographical focus (Figure 3). The six largest museum collections contributed almost 60% of pre-1950 museum records, but they contributed only 22% of the post-1950 records, presumably because many of the other collections were established after this date. These smaller collections lacked data from Eastern Europe and Northern Asia but showed a greater density of records from within Western Europe than the top six collections. A nestedness analysis [19] showed that small collections tended to house only the most frequently collected species (matrix temperature  = 4.12°C, P<0.001).

Museum collection data are of particular value due to their long history, their broad taxonomic and geographic span, and the concentration of specimens and the expertise available. However, museum collecting has declined markedly whereas information from the only other early historical source, the literature, has generally increased over time. The very recent dip in literature records is likely a transient phenomenon reflecting the time taken for observations to be published and made available.

Our study indicates that there are many historical data which could be very valuable in analysing overall changes in biodiversity, in particular in museums. On their own, the museum data present an incomplete picture, but the historical base-line they provide significantly aids interpretation of the data from other sources. Looking to the future, it is possible that trip report websites and other forms of citizen science, a rapidly growing source of species distribution data, will replace museums' roles in supplying a biodiversity base-line. However, such data are of variable quality, there is no specimen to validate the data, and birdwatchers' trips are heavily biased towards certain areas, though they do cover a wide range of species. It will be hard to compensate fully for the many unique benefits provided by museum data, especially given the extent of expert support and validation associated with them.

The different sources also contain spatial biases, with museum data being most cosmopolitan and the other sources being geographically focussed. These spatial biases are presumably not solely due to the generation of data but also to our ability to access these data. Our ringing data, for example were almost all from Western Europe. With the notable exception of the atlas data, records tend to be weighted towards sites where tourists and bird specialists visit. There are large areas of lower biodiversity habitats, places that are difficult to visit for logistical or political reasons, regions with low scientific capacity and areas where there are few rare species or protected areas where data gathering has tended to slow down considerably. This could well prejudice our ability to identify impending declines of relatively abundant species or in species-poor areas (Box 2).

**BOX 2. Three Examples of How Bias in Species Occurrence Data Can Be Misleading**

1. Different data sources may focus on different areas of a species' range, as illustrated in Figure 6, by the literature and museum records of the red junglefowl (*Gallus gallus*). Sole reliance on the literature records would result in almost all of the Philippine population being overlooked. Indeed, this seems to have been the case for much of the twentieth century, it being widely believed that the species was not native to the Philippines. However, phenotypic and geographic evidence, largely collected from museum collections, suggests that the junglefowl is indigenous to the archipelago [30].
2. The recent focus on threatened species has led to the discovery of new populations. This may lead to the illusion that a species' range has expanded over time (Figure 7), as illustrated for the Hainan hill-partridge (*Arborophila ardens*) and Cabot's tragopan (*Tragopan caboti*) where the range is inferred from pre-1930 and post-1990 data. Even using all of our data sources it will be extremely difficult for us to uncover how the true ranges of these threatened species have changed over time.
3. Studies of biodiversity trends in data-rich and data-poor regions could give very different results. A comparison of pre-1930 and post-1990 data for all species across the Indian Subcontinent (Figure 8) suggests quite severe biodiversity loss from the central plains, for example, but not from the Himalayas. However, this may simply be sampling artefact. Ornithologists no longer tend to visit low-biodiversity areas, making it difficult for us to infer these regions' biodiversity status. To understand the true picture we must control for sampling effort, perhaps by using records of an abundant species that we know not to have declined.

In summary, museum data provided the most comprehensive historical coverage, although were time-consuming to bring together. Literature records have increased in their number and coverage through time, whereas ringing, atlas, and website data are almost exclusively restricted to recent decades. Other than museum records, which had reasonably even spatial coverage across the entire study region, most records were biased towards Western Europe and Southeast Asia.

## Sampling Biases Relative to Conservation Status

**Figure 4:**
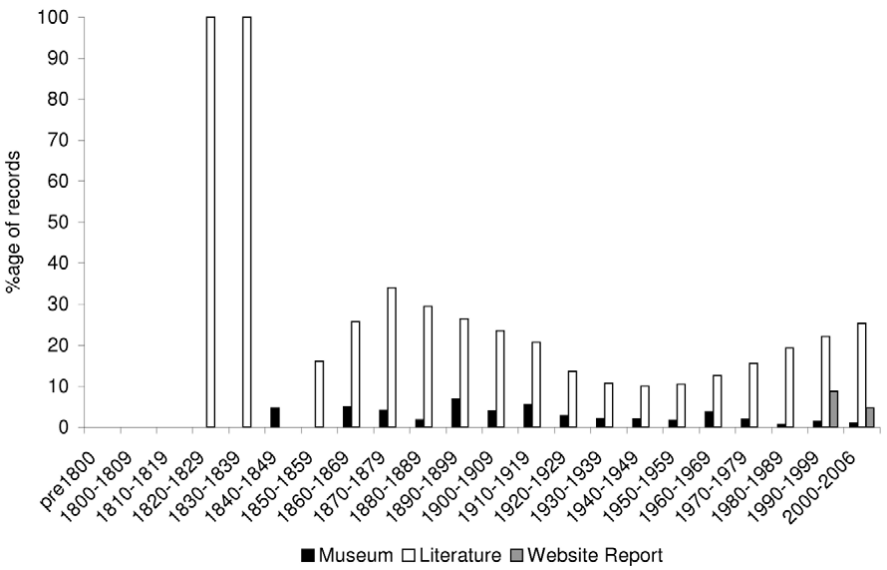
Percentage of records by source relating to species that are currently designated as threatened. For reference, 30% of the species in the database are currently threatened.

We identified the conservation status of each species in our dataset using the 2008 IUCN Red List (www.iucnredlist.org). Species in the categories Critically Endangered, Endangered, and Vulnerable are considered “threatened”; the rest are considered “non-threatened” Using these data we find that the proportion of currently threatened species sampled in our database changes with time (Figure 4). Threatened species now represent about 30% of the galliform species in our study group but, as they are often less detectable, have smaller geographic ranges, and are found at much lower abundances than their more common counterparts, we would not expect them to have made up 30% of the records in the database. Indeed, threatened species accounted for only 3% of the museum records and 5% of website report records. No threatened species were recorded by the atlas or ringing data, presumably because these survey methods are biased away from places where threatened species occur. In contrast, threatened species featured much more prominently in the literature accounting for almost 20% of records, a proportion far greater than their abundance relative to non threatened species. The proportion of literature records relating to threatened species showed a decline from the 1870s to the 1940s followed by a sharp rise from 1960 to the present day, presumably reflecting current conservation interests and a changing focus of scientific field studies. This trend is due to the identity of the species being studied, and is not necessarily attributable to a changing frequency of threat. Threatened species were first recorded in website trip reports in 1998 and account for only 5% of their records. These more species-representative reports may therefore prove extremely important in the future in informing us on a baseline of non-threatened biodiversity—a role that museums no longer play.

**Figure 5:**
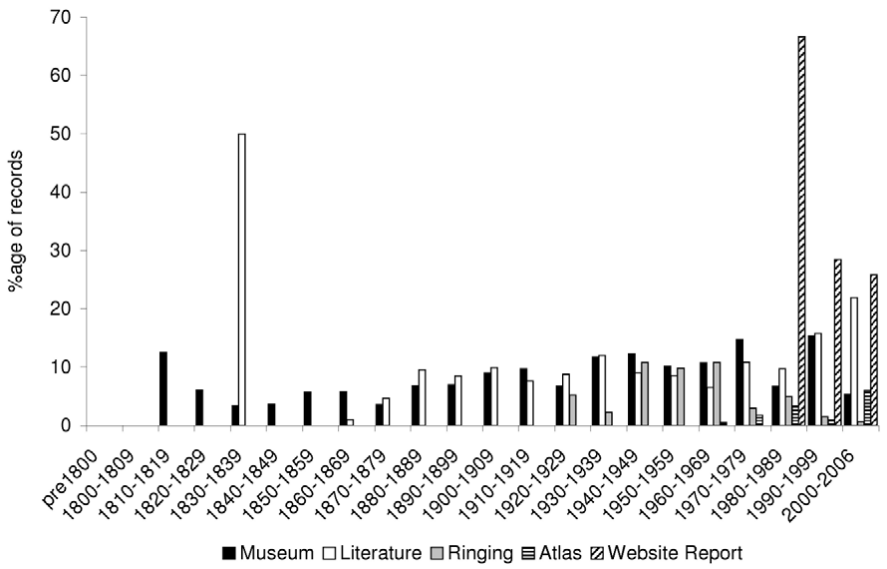
Percentage of records by source from protected areas currently designated as IUCN I-IV.

We also recorded whether locations lay in the area of currently protected habitat, defined as being classified by IUCN as areas in Categories IUCN I-IV [20]. Approximately 4% of the land area within the Palaearctic and Indo-Malay realms is protected according to this definition and should therefore be under management primarily for the conservation of biodiversity. The proportion of records from within these protected areas has consistently increased over time, perhaps reflecting habitat clearance in unprotected landscapes, but also the increasing emphasis on protected areas as a conservation tool (Figure 5). Perhaps not surprisingly, since these are popular destinations for birders, the website trip report data have the highest proportion of records from within protected areas (26%), and the proportion of museum and literature records also exceeds random expectation (9% and 13%, respectively).

**Figure 6:**
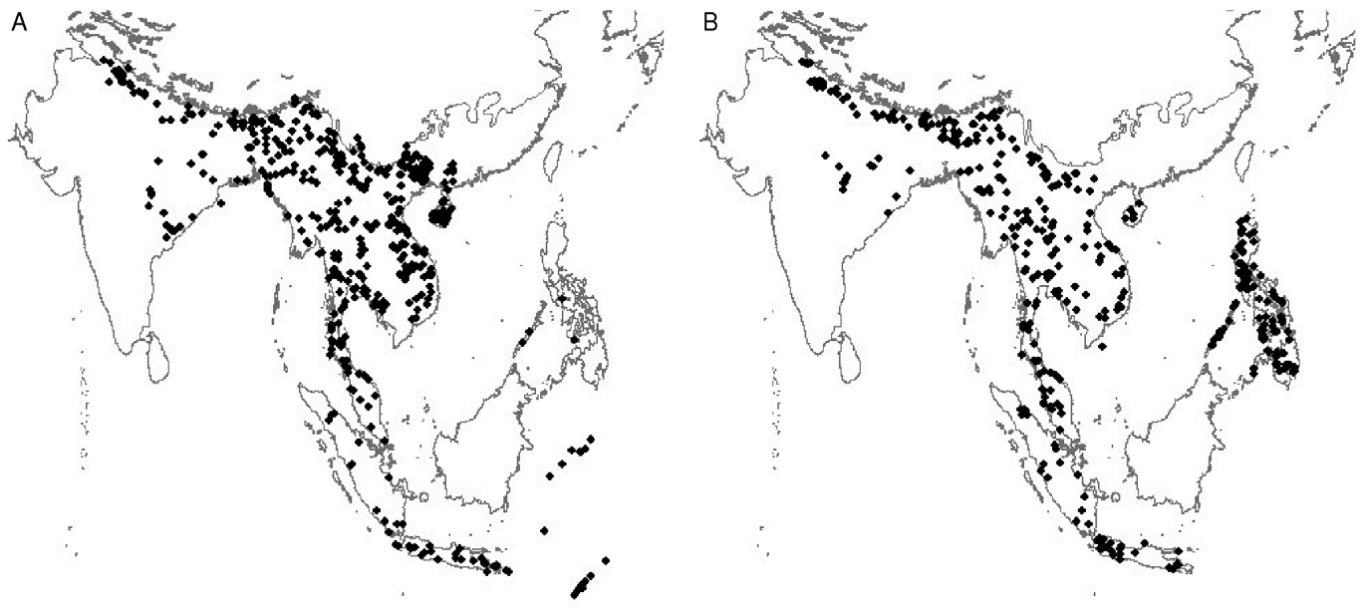
Records of the red junglefowl (*Gallus gallus*) from A) the literature and B) museum collections.

**Figure 7:**
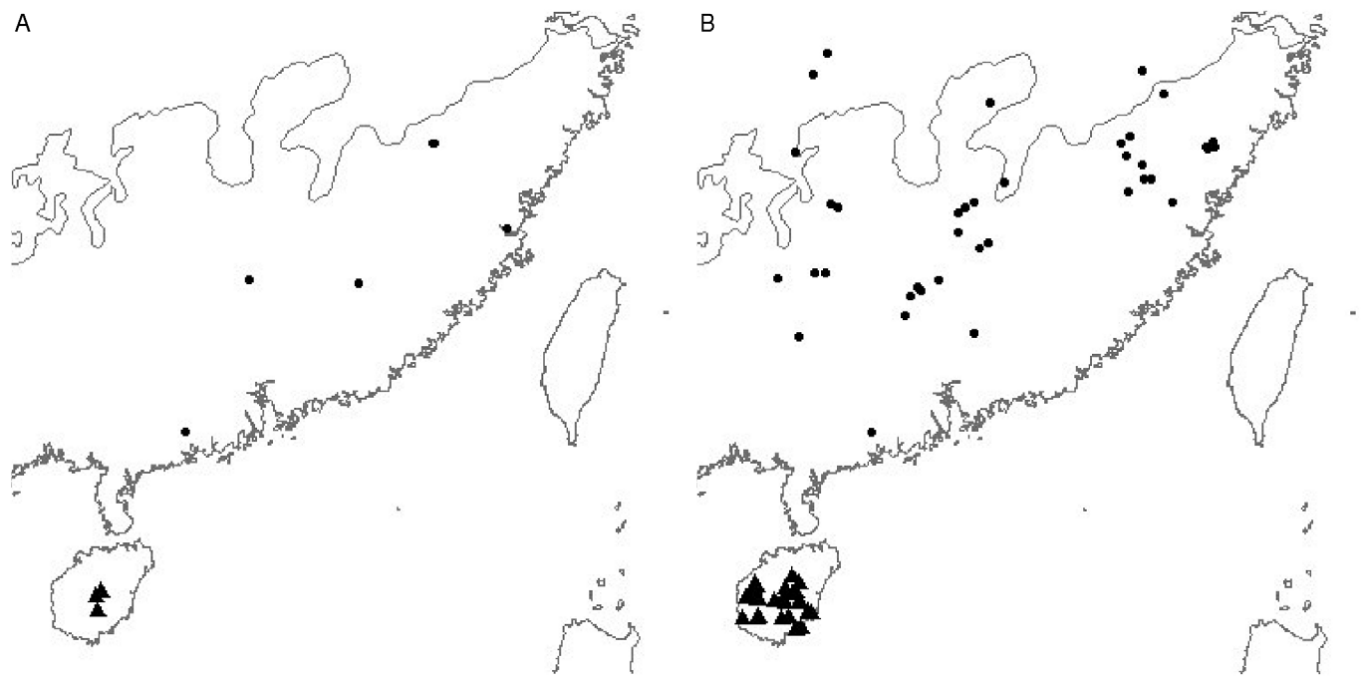
Records for the Hainan hill-partridge (*Arborophila ardens*) (triangle) and Cabot's tragopan (*Tragopan caboti*) (circle) from A) pre-1930 and B) 1990–2006.

**Figure 8:**
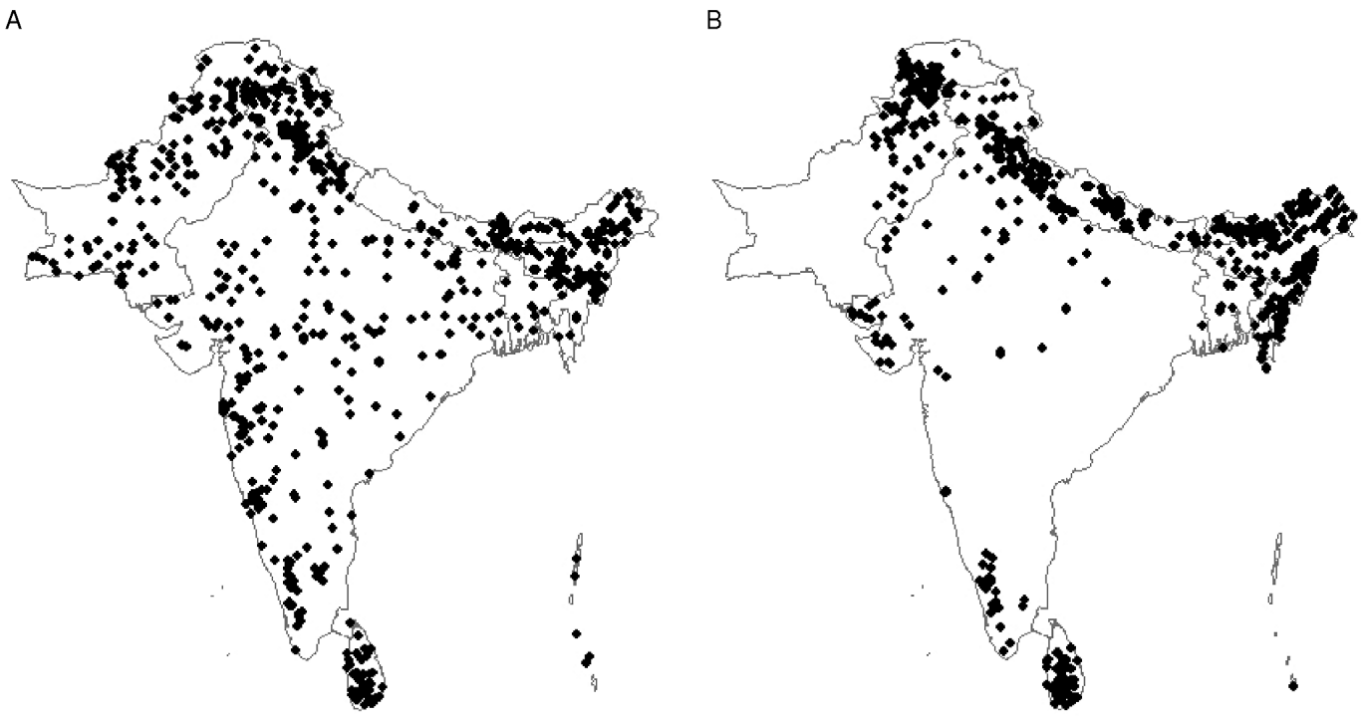
Records for all galliform species across the Indian Subcontinent from A) pre-1930 and B) 1990–2006.

Whilst the focus on threatened species and protected areas is understandable, the resulting biases must be taken into account when identifying changes in biodiversity over time. For example, a change in the number of records of threatened species results from some combination of changes in sampling patterns and genuine change in the population sizes or distributions of threatened species. Also, the paucity of information relating to non-threatened species and areas of low species richness could make it much harder to detect future changes (Box 2).

## Data Availability

This study raises several general issues about the archiving and availability of species' distribution data, especially of historical records. The world's museums contain information that is irreplaceable, especially a unique historical perspective, yet their information is often hard to access, even in the case of some well-funded, national museums. Different museums focus on different geographical areas, and therefore the maintenance of the collections and records is potentially vulnerable to local political and economic pressures, which may or may not value the collections enough to invest sufficiently in them. During data collection, we observed many older museum specimens and their labels deteriorating, and noted that often interpretation requires specialist knowledge that may reside solely with individual museum curators. Other issues of concern included poorly documented specimens, hard-to-read handwritten labels, and place names that cannot be found in current atlases and gazetteers. Databasing and, if possible, georeferencing the information in these collections is a priority to ensure that these unique and valuable data are preserved for the future.

The other source that was valuable in historical terms was the literature. We had access to a wide variety of scientific journals through major zoological libraries in London, Oxford, Cambridge, and Beijing. This information would be hard for many people to access. We also visited specialist collections and used the grey literature extensively. In fact only a small proportion of records came from scientific journals (See Figure S1) and the grey literature turned out to be extremely important and informative. This raises the question of how long grey literature may remain accessible; many recent key species and/or regional reports are already very difficult to obtain. Once more, online databasing of observations from field studies would not only ease data gathering but could help guarantee the data's longevity. Initiatives such as Morpho [http://knb.ecoinformatics.org/morphoportal.jsp], Ecological Archives [http://esapubs.org/Archive/], and Dryad [http://datadryad.org/repo] (where the data in this study are published) should mean that literature data become easier to gather and maintain in the future. Efforts being made by GBIF to extend their data gathering, and new initiatives such as GEO BON [21] are also important. On a similar note, we would advocate a general practice to database ringing records, as exemplified by the European Union for Bird Ringing (Euring) [www.euring.org].

Website trip reports, a newer data source, are of course far more accessible although their use could be greatly improved were more of their data georeferenced. Harnessing citizen science to monitor the world's biodiversity in this kind of way is becoming a real possibility [22],[23]. The development of the Internet and mobile computing has led to a vast increase in citizen science projects, which can facilitate collection and distribution of all kinds of taxonomic data from a wide geographic area at minimal cost. In any databasing venture, it should be remembered that descriptions of geographic localities are often useless if co-ordinates are not given. Online providers of aggregated data need also to provide mechanisms by which errors can be flagged and corrected [12] and data easily accessed. The eBird project (https://www.ebird.org, [23]) exemplifies this; users can only enter data that have associated dates and localities, automated checks flag up unusual sightings that are sent for checking by regional editors, and data are fed into larger global biodiversity initiatives such as GBIF. Additionally, eBird highlights areas with poor data coverage, encouraging users to enter sightings from these places. There are many other citizen science projects up and running [22],[24], and we urge further research into the potential of such initiatives in monitoring trends in biodiversity. One danger is that if too many similar projects are set up, data may be lost amongst a multitude of separate databases. Another challenge will be to ensure citizen science is able to cover all areas of the globe, in particular the species-rich tropics.

In our study we used a very well-studied group of birds and so constructing a database of sightings for all European and Asian species over a period of two centuries, although a considerable task, was probably far easier than for other taxa. Of course, historical gaps in data collection can never be filled nor the role of the museum replaced, but there are numerous examples of citizen science projects recording less charismatic taxa ranging from freshwater sponges to lichens ([22], http://www.birds.cornell.edu/citscitoolkit), and these give reason for real hope that we can eventually establish a robust mechanism for monitoring changes in global biodiversity.

## Conclusions

Knowledge derived from site-based observations and collections is biased according to the data source, location, and time period of collection. Compensating for these biases will be important for any study aiming to draw conclusions about real trends in biodiversity over time and space. Accounting for biases in biodiversity samples depends on a clear knowledge of the source and nature of those biases, and will require the development of new qualitative and quantitative methods (Box 3). It is clear that all the data sources we used are changing in their focus and frequency of reporting but that much can be learned from these data providing these patterns are understood and accounted for. An understanding of the process of—and reasons for—data generation is necessary in order to interpret each source appropriately; for example, early naturalists were interested in collecting specimens from a wide spectrum of biodiversity, whereas the current conservation focus has meant attention is biased towards threatened species and protected areas. The most complete picture of biodiversity change will be gained through building composite data sets that draw from a range of sources, perhaps in combination with models incorporating evidence-based changes [25],[26]. Despite this, museums remain an irreplaceable source of high quality historical data, and it is vital that we do not lose the base-line of biodiversity data they have supplied. Currently, trip report websites appear to offer the best hope of replacing this role, although their data cannot be re-visited and re-verified. Our explorations of primary data have led us to the following three conclusions:

1. It is critical that museum and literature data are safeguarded through databasing. While many initiatives to facilitate this are in place, the necessary financial resources to complete such an immense task frequently are not.
2. Records that are not georeferenced, dated, and fed into a centralised database have little future scientific value. All data gathering ventures should consider how their data could contribute towards establishing biodiversity baselines.
3. Data gathering must target species and areas of little or unknown conservation value as well as focusing on threatened species. Strengthening citizen science initiatives and methodologies could facilitate this.

**BOX 3. Potential Approaches for Dealing with Bias in Species Occurrence Data**

1. If the biases in species occurrence data are understood, analyses could explicitly take them into account and the excess discounted; for example, if recent literature data are known to oversample threatened species. The relative effort devoted to searching for literature and museum data could also be adjusted according to the requirements of the particular research question, cognisant of the biases inherent in data from different sources. This approach could be developed and formalised as a model of the observation process that is used to adjust results from the dataset.
2. Within standardised surveys, rarefaction methods [31] are commonly used to check whether a particular location has been sufficiently well sampled to form part of a dataset for analysis. However, owing to the variety of data types comprising many distributional datasets, survey method is far from standardised, and sampling units will frequently not be equivalent. Methods for dealing with this issue need to be developed. A species accumulation curve reaching an asymptote might not constitute evidence of sufficient sampling if data have only been collected from a small range of sources. A solution might be to weight records by their type, or to develop quantitative criteria for the proportions of records required from different sources.
3. Methods to detect biodiversity change that are more robust to variation in search effort should be used. For example, assessments of changes in occupancy will be more robust to variation in survey method and intensity than many measures of changes in abundance. Measures of determining relative, rather than absolute, change in distributions might also be preferred in situations where sampling methods have changed across time [32].

Measuring progress toward solving the global biodiversity crisis depends on credible and comparable underlying data on biodiversity through time [9]. We have shown that even primary sources of biodiversity information are subject to a range of biases that fundamentally affect their interpretation and therefore their reliability in measuring biodiversity change. The development of methods to deal with these challenges is urgently required, and this essay is intended to serve as a starting point.

## Supporting Information

Figure S1 **The numbers of journal records by decade taken from JCR (2007) listed and non-listed journals.** The number of records for 2000–2006 has been extrapolated to 2000–2009 for ease of comparison with the other decades.
(0.12 MB TIF)

Table S1 **Species list.** Threat-listings in italics indicate species which are not recognised by the IUCN. In these instances, ratings are taken from Madge and McGowan 2002, Pheasants, partridges, and grouse.
(0.14 MB DOC)

Table S2 **The responses of museums to enquiries for species distribution data.** Requests for information went to 338 museums. Museums may have given more than one response if, for example, part of their collections are catalogued electronically and part on paper.
(0.03 MB DOC)

Table S3 **The museum collections from which we were able to obtain data.**
(0.12 MB DOC)

Table S4 **Percentage of dated records which could be georeferenced A) per data source and B) per time period.** Also given is the proportion of records of known accuracy where the georeferenced location was accurate to within 10 minutes. Atlas data are excluded since all atlas records were georeferenced.
(0.03 MB DOC)

Text S1 **Ringing groups from which we were able to obtain data.**
(0.02 MB DOC)

Text S2 **The atlases from which we digitised records.**
(0.03 MB DOC)

Dataset S1 **GALLIFORM: WPA Eurasian Database v 1.0.** The records which could be at least approximately dated and georeferenced are contained in a comma delimited text file. The file gives information on the data source, the year of the record, the species the record relates to, the threat status of the species, the country the record is from and whether the record came from inside a protected area. The column names are mostly self-explanatory. In cases where an exact year of record was not known, a date range is given. For example, “Pre 1980” would indicate a record from 1979 or earlier. Similarly, “Post 1980” would indicate a record from 1981 or later. For the column “Threatened?,” “0” indicates a non-threatened species and “1” a threatened species. For the column “Inside a Protected Area?”, “0” indicates the record is from outside a protected area and “1” inside a protected area.
(8.47 MB TXT)

## References

[1] UNEP (United Nations Environment Programme) (2002). Report on the sixth meeting of the Conference of the Parties to the Convention on Biological Diversity (UNEP/CBD/COP/20/Part 2) Strategic Plan Decision VI/26 in CBD..

[2] Balmford A, Bennun L, ten Brink B, Cooper D, Cote I. M (2005). The convention on biological diversity's 2010 target.. Science.

[3] Collen B, Loh J, Whitmee S, McRae L, Amin R (2009). Monitoring Change in Vertebrate Abundance: the Living Planet Index.. Conserv Biol.

[4] Butchart S. H. M, Stattersfield A. J, Baillie J. E. M, Bennun L. A, Stuart S. N (2005). Using Red List Indices to measure progress towards the 2010 target and beyond.. Philos Trans R Soc Lond B.

[5] Sheppard C (1995). The Shifting Baseline Syndrome.. Mar Pollut Bull.

[6] Willis K. J, Araújo M. B, Bennett K. D, Figueroa-Rangel B, Froyd C. A (2007). How can a knowledge of the past help to conserve the future? Biodiversity conservation and the relevance of long-term ecological studies.. Philos Trans R Soc Lond B.

[7] Balmford A (1999). (Less and less) great expectations.. Oryx.

[8] Butchart S. H. M, Stattersfield A. J, Bennun L. A, Shutes S. M, Akcakaya H. R (2004). Measuring global trends in the status of biodiversity: Red List Indices for birds.. PLoS Biology.

[9] Soberón J, Peterson A. T (2009). Monitoring biodiversity loss with primary species-occurrence data: Toward national-level indicators for the 2010 target of the Convention on Biological Diversity.. Ambio.

[10] Collen B, Ram M, Zamin T, McRae L (2008). The tropical biodiversity data gap: addressing disparity in global monitoring.. Tropical Conservation Science.

[11] Edwards J. L (2004). Research and societal benefits of the Global Biodiversity Information Facility.. Bioscience.

[12] Robertson D. R (2008). Global biogeographical data bases on marine fishes: caveat emptor.. Divers Distrib.

[13] Yesson C, Brewer P. W, Sutton T, Caithness N, Pahwa J. S (2007). How global is the global biodiversity information facility?. PLoS ONE.

[14] Loh J, Green R. E, Ricketts T, Lamoreux J, Jenkins M (2005). The Living Planet Index: using species population time series to track trends in biodiversity.. Philos Trans R Soc Lond B Biol Sci.

[15] McGowan P. J. K, del Hoyo J, Elliott A, Sargatal J (1994). Family Phasianidae (Pheasants and Partridges).. Handbook of the Birds of the World.

[16] McGowan P. J. K, Ding C-Q, Kaul R (1999). Protected areas and the conservation of grouse, partridges and pheasants in East Asia.. Anim Conserv.

[17] Moerman D. E, Estabrook G. F (2006). The botanist effect: counties with maximal species richness tend to be home to universities and botanists.. J Biogeogr.

[18] Pautasso M, McKinney M. L (2007). The botanist effect revisited: Plant species richness, county area and human population size in the United States.. Conserv Biol.

[19] Atmar W, Patterson B. D (1995). The nestedness temperature calculator: a visual basic program, including 294 presence-absence matrices.. AICS Research Inc., University Park, NM and The Field Museum, Chicago, IL.

[20] IUCN (1994). Guidelines for Protected Area Management Categories..

[21] Scholes R. J, Mace G. M, Turner W, Geller G. N, Jürgens N (2008). Toward a global biodiversity observing system.. Science.

[22] Silvertown J (2009). A new dawn for citizen science.. Trends in Ecol Evol.

[23] Sullivan B. L, Wood C. L, Iliff M. J, Bonney R. E, Fink D (2009). eBird: A citizen-based bird observation network in the biological sciences.. Biol Conserv.

[24] Cohn J. P (2008). Citizen science: Can volunteers do real research?. Bioscience.

[25] Cowling R. M, Knight A. T, Faith D. P, Ferrier S, Lombard A. T (2004). Nature conservation requires more than a passion for species.. Conserv Biol.

[26] Graham C. H, Ferrier S, Huettman F, Moritz C, Peterson A. T (2004). New developments in museum-based informatics and applications in biodiversity analysis.. Trends in Ecol Evol.

[27] Roselaar C. S (2003). An inventory of major European bird collections. Bull Br Ornitho.. Club.

[28] McGowan P. J. K (1996). Mapping the distribution of Asian partridges and pheasants as a requirement for identifying conservation priorities.. Acta Zoological Sinica.

[29] UNEP-WCMC (2006). World Database on Protected Areas (WDPA). 2006 ed.

[30] Parkes K. C (1962). The red junglefowl of the Philippines - native or introduced?. Auk.

[31] Colwell R. K, Coddington J. A (1994). Estimating terrestrial biodiversity through extrapolation.. Philos Trans R Soc Lond B.

[32] Telfer M. G, Preston C. D, Rothery P (2002). A general method for measuring relative change in range size from biological atlas data.. Biol Conserv.

