# Supplementary figures and images for: Distorted Views of Biodiversity: Spatial and Temporal Bias in Species Occurrence Data
Source: PLoS Biol. 2010 Jun 1;8(6):e1000385. doi: 10.1371/journal.pbio.1000385 (PMC2879389; doi:10.1371/journal.pbio.1000385)

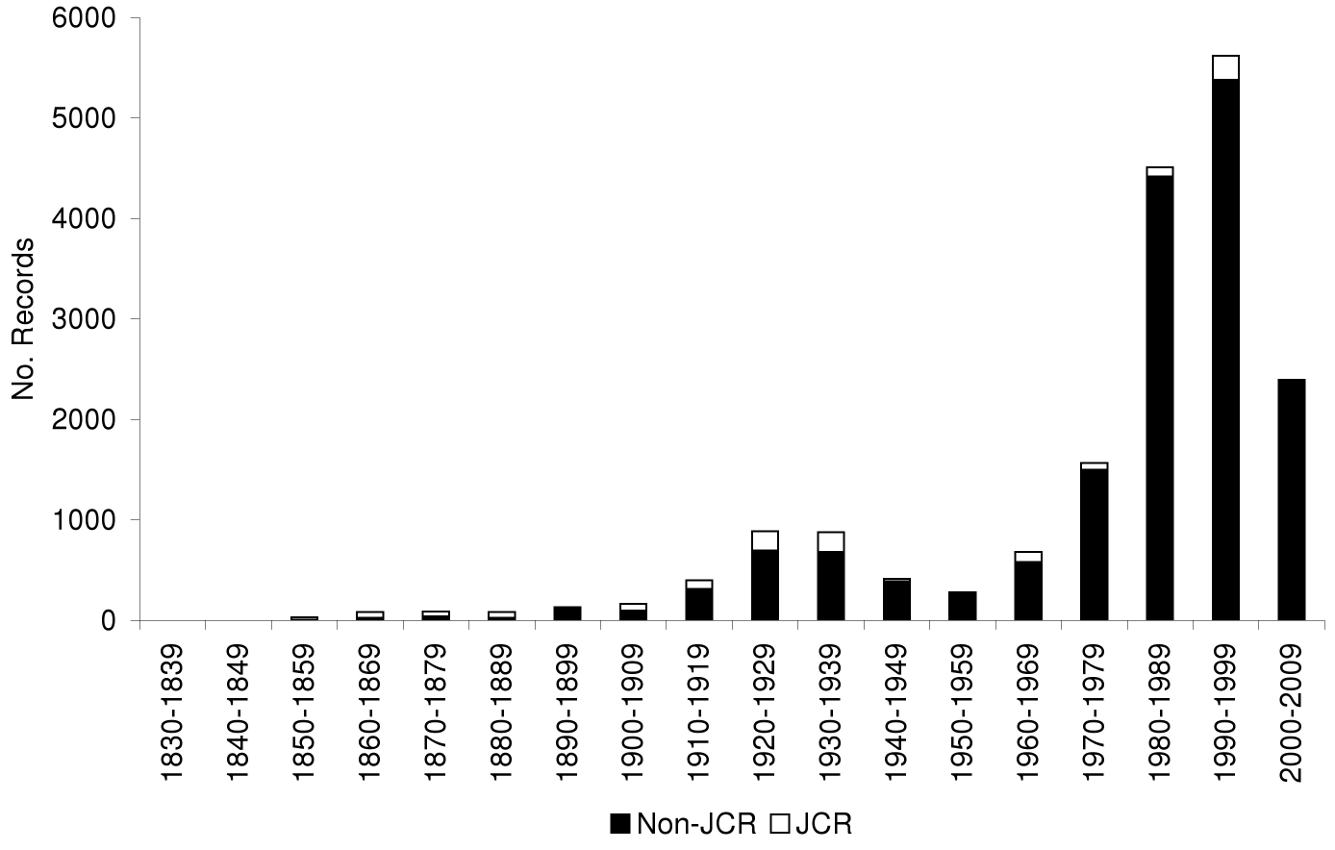

Supplement: Figure S1 — The numbers of journal records by decade taken from JCR (2007) listed and non-listed journals. The number of records for 2000–2006 has been extrapolated to 2000–2009 for ease of comparison with the other decades. (0.12 MB TIF) [file pbio.1000385.s001.tif]
