# Supplementary material for: Distorted Views of Biodiversity: Spatial and Temporal Bias in Species Occurrence Data
Source: PLoS Biol. 2010 Jun 1;8(6):e1000385. doi: 10.1371/journal.pbio.1000385 (PMC2879389; doi:10.1371/journal.pbio.1000385)
Supplement: Table S1 — Species list. Threat-listings in italics indicate species which are not recognised by the IUCN. In these instances, ratings are taken from Madge and McGowan 2002, Pheasants, partridges, and grouse. (0.14 MB DOC) [file pbio.1000385.s002.doc]

Table S1. Species list. Threat-listings in italics indicate species which are not recognised by the IUCN. In these instances, ratings are taken from Madge & McGowan 2002, Pheasants, partridges and grouse.

| English name | Scientific name | 2008 IUCN Threat-listing |
| --- | --- | --- |
| Snow partridge | *Lerwa lerwa* | Least concern |
| See-see partridge | *Ammoperdix griseogularis* | Least concern |
| Sand partridge | *Ammoperdix heyi* | Least concern |
| Caucasian snowcock | *Tetraogallus caucasicus* | Least concern |
| Caspian snowcock | *Tetraogallus caspius* | Least concern |
| Altai snowcock | *Tetraogallus altaicus* | Least concern |
| Tibetan snowcock | *Tetraogallus tibetanus* | Least concern |
| Himalayan snowcock | *Tetraogallus himalayensis* | Least concern |
| Verreaux's monal-partridge | *Tetraophasis obscurus* | Least concern |
| Szechenyi's monal-partridge | *Tetraophasis szechenyii* | Least concern |
| Rock partridge | *Alectoris graeca* | Least concern |
| Chukar | *Alectoris chukar* | Least concern |
| Przevalski's partridge | *Alectoris magna* | Least concern |
| Philby's partridge | *Alectoris philbyi* | Least concern |
| Barbary partridge | *Alectoris barbara* | Least concern |
| Arabian partrridge | *Alectoris melanocephala* | Least concern |
| Red-legged partridge | *Alectoris rufa* | Least concern |
| Grey francolin | *Francolinus pondicerianus* | Least concern |
| Swamp francolin | *Francolinus gularis* | Vulnerable |
| Black francolin | *Francolinus francolinus* | Least concern |
| Painted francolin | *Francolinus pictus* | Least concern |
| Chinese francolin | *Francolinus pintadeanus* | Least concern |
| Grey partridge | *Perdix perdix* | Least concern |
| Daurian partridge | *Perdix dauurica* | Least concern |
| Tibetan partridge | *Perdix hodgsoniae* | Least concern |
| Long-billed partridge | *Rhizothera longirostris* | Near threatened |
| Black wood-partrridge | *Melanoperdix nigra* | Vulnerable |
| Common quail | *Coturnix coturnix* | Least concern |
| Japanese quail | *Coturnix japonica* | Least concern |
| Rain quail | *Coturnix coromandelica* | Least concern |
| King quail | *Coturnix chinensis* | Least concern |
| Jungle bush-quail | *Perdicula asiatica* | Least concern |
| Rock bush-quail | *Perdicula argoondah* | Least concern |
| Painted bush-quail | *Perdicula erythrorhyncha* | Least concern |
| Manipur bush-quail | *Perdicula manipurensis* | Vulnerable |
| Himalayan quail | *Ophrysia superciliosa* | Critically endangered |
| Mountain bamboo-partridge | *Bambusicola fytchii* | Least concern |
| Chinese bamboo-partridge | *Bambusicola thoracica* | Least concern |
| Necklaced hill-partridge | *Arborophila torqueola* | Least concern |
| Taiwan hill-partridge | *Arborophila crudigularis* | Near threatened |
| White-cheeked hill-partridge | *Arborophila atrogularis* | Near threatened |
| Hainan hill-partridge | *Arborophila ardens* | Vulnerable |
| Orange-necked hill-partridge | *Arborophila davidii* | Endangered |
| Cambodian hill-partridge | *Arborophila cambodiana* | Vulnerable |
| Siamese hill-partridge | *Arborophila diversa* | Vulnerable |
| Red-billed hill-partridge | *Arborophila rubrirostris* | Least concern |
| Bornean hill-partridge | *Arborophila hyperythra* | Least concern |
| Brown-breasted hill-partridge | *Arborophila brunneopectus* | Least concern |
| Grey-breasted hill-partridge | *Arborophila orientalis* | Vulnerable |
| Sumatran hill-partridge | *Arborophila sumatrana* | Least concern |
| Malaysian hill-partridge | *Arborophila campbelli* | Vulnerable |
| Javan hill-partridge | *Arborophila javanica* | Least concern |
| Rufous-throated hill-partridge | *Arborophila rufogularis* | Least concern |
| Chestnut-breasted hill-partridge | *Arborophila mandellii* | Vulnerable |
| Collared hill-partridge | *Arborophila gingica* | Vulnerable |
| Sichuan hill-partridge | *Arborophila rufipectus* | Endangered |
| Chestnut-necklaced hill-partridge | *Arborophila charltonii* | Near threatened |
| Scaly-breasted hill-partridge | *Arborophila chloropus* | Least concern |
| Annam hill-partridge | *Arborophila merlini* | Endangered |
| Ferruginous partridge | *Caloperdix oculea* | Near threatened |
| Crimson-headed partridge | *Haematortyx sanguiniceps* | Least concern |
| Crested partridge | *Rollulus rouloul* | Near threatened |
| Red spurfowl | *Galloperdix spadicea* | Least concern |
| Painted spurfowl | *Galloperdix lunulata* | Least concern |
| Sri Lanka spurfowl | *Galloperdix bicalcarata* | Least concern |
| Blood pheasant | *Ithaginis cruentus* | Least concern |
| Temminck's tragopan | *Tragopan temminckii* | Least concern |
| Cabot's tragopan | *Tragopan caboti* | Vulnerable |
| Western tragopan | *Tragopan melanocephalus* | Vulnerable |
| Satyr tragopan | *Tragopan satyra* | Near threatened |
| Blyth's tragopan | *Tragopan blythii* | Vulnerable |
| Himalayan monal | *Lophophorus impejanus* | Least concern |
| Sclater's monal | *Lophophorus sclateri* | Vulnerable |
| Chinese monal | *Lophophorus lhuysii* | Vulnerable |
| Red junglefowl | *Gallus gallus* | Least concern |
| Grey junglefowl | *Gallus sonneratii* | Least concern |
| Sri Lanka junglefowl | *Gallus lafayettii* | Least concern |
| Green junglefowl | *Gallus varius* | Least concern |
| Edward's pheasant | *Lophura edwardsi* | Endangered |
| Vietnamese pheasant | *Lophura hatinhensis* | Endangered |
| Swinhoe's pheasant | *Lophura swinhoii* | Vulnerable |
| Kalij pheasant | *Lophura leucomelanos* | Least concern |
| Silver pheasant | *Lophura nycthemera* | Least concern |
| Hoogerwerf's pheasant | *Lohpura hoogerwerfi* | Vulnerable |
| Salvadori's pheasant | *Lohpura inornata* | Vulnerable |
| Crestless fireback | *Lophura erythropthalma* | Vulnerable |
| Crested fireback | *Lophura ignita* | Near threatened |
| Reeve's pheasant | *Syrmaticus reevesii* | Vulnerable |
| Siamese fireback | *Lohpura diardi* | Near threatened |
| Bulwer's pheasant | *Lophura bulweri* | Vulnerable |
| Tibetan eared-pheasant | *Crossoptilon harmani* | Near threatened |
| White eared-pheasant | *Crossoptilon crossoptilon* | Near threatened |
| Blue eared-pheasant | *Crossoptilon auritum* | Least concern |
| Brown eared-pheasant | *Crossoptilon mantichuricum* | Vulnerable |
| Koklass pheasant | *Pucrasia macrolopha* | Least concern |
| Cheer pheasant | *Catreus wallichii* | Vulnerable |
| Mrs Hume's pheasant | *Syrmaticus humiae* | Near threatened |
| Elliot's pheasant | *Syrmaticus ellioti* | Vulnerable |
| Mikado pheasant | *Syrmaticus mikado* | Near threatened |
| Copper pheasant | *Syrmaticus soemmerringii* | Near threatened |
| Common pheasant | *Phasianus colchicus* | Least concern |
| Green pheasant | *Phasianus versicolor* | *Least concern* |
| Golden pheasant | *Chrysolophus pictus* | Least concern |
| Lady Amherst's pheasant | *Chrysolophus amherstiae* | Least concern |
| Sumatran peacock-pheasant | *Polyplectron chalcurum* | Least concern |
| Mountain peacock-pheasant | *Polyplectron inopinatum* | Vulnerable |
| Bornean peacock-pheasant | *Polyplectron schleiermacheri* | Endangered |
| Palawan peacock-pheasant | *Polyplectron emphanum* | Vulnerable |
| Hainan peacock-pheasant | *Polyplectron katsumatae* | *Endangered* |
| Grey peacock-pheasant | *Polyplectron bicalcaratum* | Least concern |
| Germain's peacock-pheasant | *Polyplectron germaini* | Near threatened |
| Malayan peacock-pheasant | *Polyplectron malacense* | Vulnerable |
| Crested argus | *Rheinardia ocellata* | Near threatened |
| Great argus | *Argusianus argus* | Near threatened |
| Indian peafowl | *Pavo cristatus* | Least concern |
| Green peafowl | *Pavo muticus* | Vulnerable |
| Siberian spruce grouse | *Falcipennis falcipennis* | Near threatened |
| Red grouse | *Lagopus lagopus* | Least concern |
| Rock ptarmigan | *Lagopus mutus* | Least concern |
| Black grouse | *Lyrurus tetrix* | Least concern |
| Caucasian grouse | *Lyrurus mlokosiewiczi* | Near threatened |
| Western capercaillie | *Tetrao urogallus* | Least concern |
| Black-billed capercaillie | *Tetrao parvirostris* | Least concern |
| Hazel grouse | *Tetrastes bonasia* | Least concern |
| Severtzov's grouse | *Tetrastes sewerzowi* | Near threatened |
| Philippine megapode | *Megapodius cumingi* | Least concern |
| Nicobar megapode | *Megapodius nicobariensis* | Vulnerable |
