# Supplementary material for: Distorted Views of Biodiversity: Spatial and Temporal Bias in Species Occurrence Data
Source: PLoS Biol. 2010 Jun 1;8(6):e1000385. doi: 10.1371/journal.pbio.1000385 (PMC2879389; doi:10.1371/journal.pbio.1000385)
Supplement: Table S2 — The responses of museums to enquiries for species distribution data. Requests for information went to 338 museums. Museums may have given more than one response if, for example, part of their collections are catalogued electronically and part on paper. (0.03 MB DOC) [file pbio.1000385.s003.doc]

Table S2. The responses of museums to enquiries for species distribution data. Requests for information went to 338 museums. Museums may have given more than one response if, for example, part of their collections are catalogued electronically and part on paper.

| Outcome | No. of museums |
| --- | --- |
| Publicly available online database | 28 |
| Electronic catalogue | 83 |
| Paper catalogue | 16 |
| Collected the data for us | 18 |
| We visited | 9 |
| Invitation to visit but visit not deemed feasible/worthwhile | 4 |
| No relevant data/unable to supply data | 63 |
| No response | 150 |
