# Supplementary material for: Distorted Views of Biodiversity: Spatial and Temporal Bias in Species Occurrence Data
Source: PLoS Biol. 2010 Jun 1;8(6):e1000385. doi: 10.1371/journal.pbio.1000385 (PMC2879389; doi:10.1371/journal.pbio.1000385)
Supplement: Table S3 — The museum collections from which we were able to obtain data. (0.12 MB DOC) [file pbio.1000385.s004.doc]

Table S3. The museum collections from which we were able to obtain data.

| Museum Title | Museum Code |
| --- | --- |
| Academy of Natural Sciences, Philadelphia, USA | ANSP |
| American Museum of Natural History, USA | AMNH |
| Auckland Museum, New Zealand | AIM |
| Beijing Institute of Zoology, China |  |
| Bell Museum of Natural History, University of Minnesota, USA | MMNH |
| Bernice P. Bishop Museum, USA | BPBM |
| Biologie Zentrum des Oberostereichisches Landesmuseums, Linz, Austria |  |
| Booth Museum of Natural History, Brighton, UK | BMBN |
| Borror Laboratory of Bioacoustics, USA | BLB |
| Bristol Museums and Art Gallery Service, UK | CMBK |
| British Library National Sound Archive (NSA), UK | NSA |
| Burke Museum of Natural History and Culture, USA | UWBM |
| Californian Academy of Sciences, USA | CAS |
| Canadian Museum of Nature, Canada | CMN |
| Carnegie Museum of Natural History, USA | CM |
| Chelmsford Museum, UK |  |
| Cleveland Museum of Natural History, UK | CMNH |
| Coll. "A. Noro", City of Graglia (Biella), Italy |  |
| Cornell University Museum of Vertebrates, USA | CUMV |
| Delaware Museum of Natural History, USA | DMNH |
| Denver Museum of Nature and Science, USA | DMNS |
| Dorman Museum, UK | MIDDM |
| Estacion Biologica de Donana, Seville, Spain | EBD |
| Field Museum of Natural History, USA | FMNH |
| Florida Museum of Natural History, USA | UF |
| Fries Natuurmuseum, Leeuwarden, Netherlands | FNM |
| Glasgow Art Gallery and Museum, UK | GMNGZ |
| Hancock Museum, UK | NEWHM |
| Humboldt State University Wildlife Museum, USA | HSUWM |
| Institut fur Vogelforschung 'Vogelwarte Helgoland', Wilhelmshaven, Germany | IfV |
| Institut Royal des Sciences Naturelles de Belgique, Belgium | IRSNB |
| Kaunas Zoological Museum, Lithuania |  |
| Leicester City Museums Service, UK | LEICT |
| Liverpool Museum, UK | LIVCM |
| Malmo Museer, Sweden |  |
| Manchester Museum, University of Manchester, UK | MMUM |
| Musee Cantonal de Zoologie, Lausanne, Switzerland | MZL |
| Musee Guimet d'Histoire Naturelle, France | MGHNL |
| Musee Zoologique de l'Universite Louis Pasteur et de la Ville de Strasbourg, France | MZS |
| Museo Civico de Storia Naturale 'Giacomo Doria', Genoa, Italy | MSNG |
| Museo Civico di Storia Naturale di Carmagnola, Italy | MCSNC |
| Museo di Storia Naturale del Mediterraneo, Livorno, Italy |  |
| Museo di Storia Naturale di Terrasini, Italy | MRST |
| Museo Municipal do Funchal, Portugal | MMF |
| Museo Nacional de Ciencias Naturales, Madrid, Spain | MNM |
| Museo Ornitologico 'F. Foschi', Italy |  |
| Museo Regionale di Scienze Naturali, Torino, Italy | MRSN |
| Museo Zoologico de La Specola, Florence, Italy | MZUF |
| Museo Zoologico dell' Accademia del Fisiocritici, Italy | MUSNAF |
| Museu Bocage, Lisbon, Portugal | BM |
| Museu de Historia Natural-Zoologia, Porto, Portugal |  |
| Museum d' Histoire Naturelle de Grenoble, France | MHNGR |
| Museum d'Histoire Naturelle de la Ville de Geneve, Switzerland | MHNG |
| Museum d'Histoire Naturelle de Neuchatel, Switzerland | MHNNL |
| Museum für Naturkunde Berlin, Germany | ZMB |
| Museum fur Naturkunde, Magdeburg, Germany | MfNM |
| Museum National d'Histoire Naturelle, Paris, France | MNHN |
| Museum of Comparative Zoology, USA | MCZ |
| Museum of Natural History and Archaeology, Trondheim, Norway |  |
| Museum of Natural History, Wroclaw University, Poland | MPUW |
| Museum of Vertebrate Zoology, USA | MVZ |
| Museum of Zoology, Bogor, Indonesia | BoM |
| Museum Victoria, Melbourne, Australia | NMVM |
| Muzeul 'Tarii Crisurilor', Oradea, Romania |  |
| Muzeum J A Komenskeho, Prerov, Czech Republic | MOSPrerov |
| National Museum of Ireland, Ireland | NMINH |
| National Museum of Natural History, Leiden, Netherlands | NNM |
| National Museum of Natural History, Smithsonian Institution, USA | USNM |
| National Museums and Galleries of Wales, UK | NMWC |
| Natural History Museum of Los Angeles County, USA | LACM |
| Natural History Musuem, Vienna, Austria | NHMV |
| Naturhistorisches Museum Bern, Switzerland | NMBE |
| Naturhistorisches Museum Mainz, Germany | NHMMZ |
| Naturhistorisches Museum, Basel, Switzerland | NHMB |
| Naturhistoriska Museet, Gothenburg, Sweden | GNM |
| Naturhistoriska Museum, Arhus, Denmark |  |
| Naturkunde Museum im Ottoneum, Kassel, Germany |  |
| North Carolina State Museum of Natural Science, USA | NCSM |
| Nottinghamshire Biological and Geological Records Centre, UK | NBGRC |
| Peabody Museum of Natural History at Yale University, USA | YPM |
| Pfalzmuseum fur Naturkunde, Bad Duerkheim, Germany | PMN |
| Plodiv Natural Science Museum, Bulgaria | NSMP |
| Regional Museum of Natural History, Bhopal, India | RMNH |
| Royal Albert Memorial Museum and Art Gallery, UK | EXEMS |
| Royal Alberta Museum, Canada | PMA |
| Ruse Natural History Museum, Bulgaria | NHMRB |
| Sam Noble Oklahoma Museum of Natural History, USA | SNOMNH |
| Santa Barbara Museum of Natural History, USA | SBMNH |
| Senckenberg Museum, Germany | SMFM |
| Shropshire County Museum Service, UK | SHRCM |
| Slater Museum of Natural History, USA | PSM |
| South African Museum, Cape Town | IZIKO |
| South Australian Museum, Australia | SAM |
| Staatliches Museum fur Naturkunde, Karlsruhe, Germany | SMNK |
| Staatliches Museum fur Naturkunde, Stuttgart, Germany | SMNS |
| Swedish Museum of Natural History, Stockholm, Sweden | NRM |
| The Herbert Museum, Coventry, UK |  |
| The Natural History Museum at Tring, UK | BMNH |
| Tullie House Museum and Art Gallery, Carlisle, UK |  |
| Uberseemuseum, Bremen, Germany | UMB |
| Ulster Museum, UK |  |
| Universita di Pavia, Italy |  |
| Universitaet Halle, Germany |  |
| Universitets Museet I Tromso, Norway | TSZV |
| University Museum of Zoology Cambridge, UK | CAMZM |
| University of Copenhagen Museum of Zoology, Denmark | ZMUC |
| University of Michigan, Museum of Zoology, USA | UMMZ |
| University of Nebraska State Museum, USA | UNSM |
| Utah Museum of Natural History, USA | UMNH |
| Vlastivedne Muzeum v Olomouci, Czech Republic | VMO |
| Westfalisches Museum fur Naturkunde, Munster, Germany | WMN |
| Zaklad Zoologii Systematycznej I Doswiadczalnej, Poland | ISEA |
| Zoological Museum Amsterdam, Netherlands | ZMA |
| Zoological Museum, Moscow, Russia | ZMMU |
| Zoological Reference Collection, Singapore | ZRC |
| Zoologicheskii Institut, St Petersburg, Russia | ZISP |
| Zoologischen Sammlung der Universitat Rostock, Germany | ZSRO |
| Zoologisches Forschungsinstitut und Museum Alexander Koenig, Germany | ZFMK |
| Zoologisches Institut und Zoologisches Museum, Hamburg, Germany | ZMH |
| Zoologisches Museum der Christian-Albrechts Universitat, Germany | ZMUK |
| Zoologisches Museum der Universitat Zurich-Irchel, Switzerland | ZMUZ |
| Zoologisk Museum, Bergen, Norway | ZMBN |
| Zoologisk Museum, Lund, Sweden | ZMUL |
| Zooloogia Muuseum, Tartu, Estonia |  |
