# Supplementary material for: Distorted Views of Biodiversity: Spatial and Temporal Bias in Species Occurrence Data
Source: PLoS Biol. 2010 Jun 1;8(6):e1000385. doi: 10.1371/journal.pbio.1000385 (PMC2879389; doi:10.1371/journal.pbio.1000385)
Supplement: Table S4 — Percentage of dated records which could be georeferenced A) per data source and B) per time period. Also given is the proportion of records of known accuracy where the georeferenced location was accurate to within 10 minutes. Atlas data are excluded since all atlas records were georeferenced. (0.03 MB DOC) [file pbio.1000385.s005.doc]

Table S4. Percentage of dated records which could be georeferenced a) per data source and b) per time period. Also given is the proportion of records of known accuracy where the georeferenced location was accurate to within 10 minutes. Atlas data are excluded since all atlas records were georeferenced.

a)

| Data source | Percentage of georeferenced records | % of known accuracy records that are accurate to within 10 minutes | Total no. records |
| --- | --- | --- | --- |
| Museum | 74% | 57% | 47972 |
| Literature | 88% | 60% | 24092 |
| Ringing | 99.9% | 99.8% | 15088 |
| Website trip report | 73% | 35% | 2223 |

b)

| Time period | Percentage of georeferenced records | % of known accuracy records that are accurate to within 10 minutes | Total no. records |
| --- | --- | --- | --- |
| 1625-1849 | 47% | 57% | 826 |
| 1850-1899 | 67% | 49% | 11123 |
| 1900-1949 | 77% | 55% | 32103 |
| 1950-2006 | 91% | 73% | 45323 |
