# Supplementary material for: Distorted Views of Biodiversity: Spatial and Temporal Bias in Species Occurrence Data
Source: PLoS Biol. 2010 Jun 1;8(6):e1000385. doi: 10.1371/journal.pbio.1000385 (PMC2879389; doi:10.1371/journal.pbio.1000385)
Supplement: Text S1 — Ringing groups from which we were able to obtain data. (0.02 MB DOC) [file pbio.1000385.s006.doc]

Text S1. Ringing groups from which we were able to obtain data.

Beringungszentrale Hiddensee

Coturnix ringing records, Italy

EURING

Finnish Museum of Natural History

Hungarian Bird Ringing Centre

National Parks Board, Singapore

Zagreb Ringing Scheme
