# Supplementary material for: Distorted Views of Biodiversity: Spatial and Temporal Bias in Species Occurrence Data
Source: PLoS Biol. 2010 Jun 1;8(6):e1000385. doi: 10.1371/journal.pbio.1000385 (PMC2879389; doi:10.1371/journal.pbio.1000385)
Supplement: Text S2 — The atlases from which we digitised records. (0.03 MB DOC) [file pbio.1000385.s007.doc]

Text S2. The atlases from which we digitised records.

1. Atlas van de Nederlandse Broedvogels 1978-1983, 1988. SOVON & Bekhuis, J.F., SOVON, Arnhem.
2. Atlas Hnizdniho Rozsireni Ptaku V CSSR 1973/77, 1987. Stastny, K., Randik, A., Hudec, K., Academia, Praha.
3. The EBCC atlas of European breeding birds: their distribution and abundance, 1997. Hagemeijer, E.J.M. & Blair, M.J., T. and A.D.Poyser, London.
4. Atlas of breeding birds of the West Midlands, 1970. Lord, J., Munns, D.J., Collins, London.
5. Eesti Linnuatlas, 1993. Renno, K.O., Valgus.
6. Atlante degli uccelli nidificanti e svernanti in Toscana, 1997. Florenzano, G.T., Arcamone, E., Baccetti, N., Meschini, E., Sposimo, P., Quad. Mus. Nat. Stor. Livorno - Monografie 1.
7. Latvian breeding bird atlas 1980-1984, 1989. Priednieks, J., Strazds, M, Strazds, A. and Petrins, A., Zinatne, Riga.
8. Atlas of breeding birds in Luxemburg, 1987. Melchior, E., Mentgen, E., Feltzer, Schmitt, R. & Weiss, J., Letzebuerger Natura Vulleschutzliga.
9. Atlas of wintering birds of Japan, 1988. Wild Bird Society of Japan, Japanese Environment Agency.
10. Atlas des oiseaux nicheurs de Belgique, 1988. Devilliers, P., Roggeman, W., Tricot, J., Del Marmol, P., Kerwijn, C., Jacob, J.P. and Anselin, A., Institut Royal des Sciences Naturelles de Belgique, Bruxelles.
11. Atlas of the breeding birds of Andorra, 2002. Thiollay, J., Associacio per a la Defensa de la Natura.
12. Zimski ornitoloski atlas Slovenije, 1994. Sovinc, A., Tehniska zalozba Slovenije, Ljubljana.
13. The atlas of breeding birds of Britain and Ireland, 1976. Sharrock, J.T.R., British Trust for Ornithology, Tring, UK.
14. Atlas das aves que nidificam em Portugal Continental, 1989. Rufino, R., SNPRCN, Portugal.
15. Birds of Moscow city and the Moscow region, 2006. Groot Koerkamp, G. and Golovach, S.I., Pensoft Pub.
16. Atlas van de Nederlandse Broedvogels 1973-1977, 1979. Texeira, R.M., Vereniging tot Behoud van Natuurmonumenten in Nederland, The Hague.
17. The new atlas of breeding birds of Britain and Ireland: 1988-1991, 1993. Gibbons, D.W., Chapman, R. and Reid, J., T. and A.D.Poyser, London.
